# Nuclear Import of UBL-Domain Protein Mdy2 Is Required for Heat-Induced Stress Response in *Saccharomyces cerevisiae*

**DOI:** 10.1371/journal.pone.0052956

**Published:** 2012-12-28

**Authors:** Khalid Arhzaouy, Massoud Ramezani-Rad

**Affiliations:** Institute for Microbiology, Heinrich Heine University, Düsseldorf, Germany; University of Kent, United Kingdom

## Abstract

Ubiquitin (Ub) and ubiquitin-like (UBL) proteins regulate a diverse array of cellular pathways through covalent as well as non-covalent interactions with target proteins. Yeast protein Mdy2 (Get5) and its human homolog GdX (Ubl4a) belong to the class of UBL proteins which do not form conjugates with other proteins. Mdy2 is required for cell survival under heat stress and for efficient mating. As part of a complex with Sgt2 and Get4 it has been implicated in the biogenesis of tail-anchored proteins. Interestingly, in response to heat stress, Mdy2 protein that is predominantly localized in the nucleus co-localized with poly(A)-binding protein Pab1 to cytoplasmic stress granules suggesting that nucleocytoplasmic shuttling is of functional importance. Here we investigate the nuclear import of Mdy2, a process that is independent of the Get4/Sgt2 complex but required for stress response. Nuclear import is mediated by an N-terminal nuclear localization signal (NLS) and this process is essential for the heat stress response. In contrast, cells expressing Mdy2 lacking a nuclear export signal (NES) behave like wild type. Importantly, both Mdy2 and Mdy2-ΔNES, but not Mdy2-ΔNLS, physically interact with Pab1 and this interaction correlates with the accumulation in cytoplasmic stress granules. Thus, the nuclear history of the UBL Mdy2 appears to be essential for its function in cytoplasmic stress granules during the rapid cellular response to heat stress.

## Introduction

Ubiquitin-like (UBL) proteins with their characteristic UBL domain are involved in a wide range of cellular processes, such as targeting and formation of nuclear compartments, spindle pole body duplication, and apoptosis. Ubiquitin-like proteins are divided into two subclasses [1]. Type-1 ubiquitin-like polypeptides (UBLs) essentially consist only of the UBL domain and function as modifiers like ubiquitin, being ligated to target proteins in a process similar to ubiquitylation. Important examples are SUMO, NEDD8, and UCRP/ISG15. In type-2 proteins the UBL domain is accompanied by other domains suggesting different functions. This is supported by the observation that type-2 proteins do not form covalent conjugates with target proteins [1], [2].

Although ubiquitin-like type-2 proteins are studied intensively little is known about their specific cellular function. *MDY2* from *Saccharomyces cerevisiae* encodes a protein of 212 amino acids containing a conserved UBL domain (residues 74–149). Unlike most yeast proteins that contain such a domain, Mdy2 does not interact with polyubiquinated proteins nor does it bind the 26S proteasome [3]. *MDY2* was identified as a gene necessary for cell survival under heat stress and for efficient mating [2], [4], [5]. The closest homolog of Mdy2 is the human ubiquitin-like protein GdX (Ubl4a) which consists of 157 amino acids [6]. The shared regions of homology show a 34% identity and encompass residues 74–212 of Mdy2 and the N-terminal 123 residues of GdX/Ubl4a. The N-terminal domain of Mdy2 is only conserved in fungi. Deletion of *MDY2* increased heat sensitivity of mutant cells. Strains contain shorter microtubules (MTs) and accumulate defects in nuclear migration during mitosis [4]. In addition, deletion of *MDY2* is associated with a five- to seven-fold reduction in mating efficiency, mainly due to defects in nuclear migration and karyogamy at the prezygotic stage [2]. Mdy2 (also known as Tma24 or Get5) was recently identified in a protein complex (composed of Sgt2, Get4 and Mdy2) implicated in tail-anchored membrane protein (TAP) insertion into the endoplasmic reticulum (ER) [7], [8], [9], [10], [11] also suggesting its important roles in the cytoplasm.

Nuclear-cytoplasmic shuttling of proteins is essential in coordinating nuclear events such as transcription and ribosome assembly with cellular processes such as translation and metabolism. It is well established that nuclear localization and export signals (NLSs and NESs) direct proteins in and out of the nucleus, respectively (reviewed in [12], [13], [14], [15], [16]). NLSs are short peptide motifs that mediate the nuclear import of proteins by binding to their receptors, known as importins (karyopherins) [12], [13], [14], [15], [16]. The best characterized transport signal is the classical NLS (cNLS) for nuclear protein import which consists of either one (monopartite) or two (bipartite) stretches of basic amino acids [17], [18], [19]. In contrast, cargos traveling toward the cytoplasm usually display a leucine-rich NES containing critical hydrophobic residues necessary for recognition by the nuclear export receptor Crm1 [20]. Nucleocytoplasmic transport proceeds through nuclear pore complexes (NPCs) that penetrate the nuclear envelope and allow passive diffusion of particles below the exclusion limit of 20–40 kDa, while larger components need to interact with a meshwork of nucleoporin repeats during translocation [21].

According to our previous studies Mdy2 localizes predominantly in the nucleus. However, under heat stress the UBL protein accumulates in cytoplasmic foci containing the poly(A)-binding protein Pab1, a marker for stress granules (SG) [4]. During glucose deprivation a minority of Mdy2 foci overlapped with processing bodies (PB) marker Dcp2, while most Mdy2 and Pab1 overlap in stress granules [4].

Here, we analyze the nucleocytoplasmic shuttling activity of Mdy2 in closer detail. We show that nuclear localization of Mdy2 is independent of Get4 and Sgt2. Furthermore, the fungal-specific N-terminus dependent nuclear import of Mdy2 is essential for the function of Mdy2 during heat stress and also, unexpectedly, for recruitment to cytoplasmic foci.

## Results and Discussion

### Analysis of Heat-induced Stress Tolerance in *mdy2, get4*, and *sgt2* Deletion Mutants

**Figure 1:**
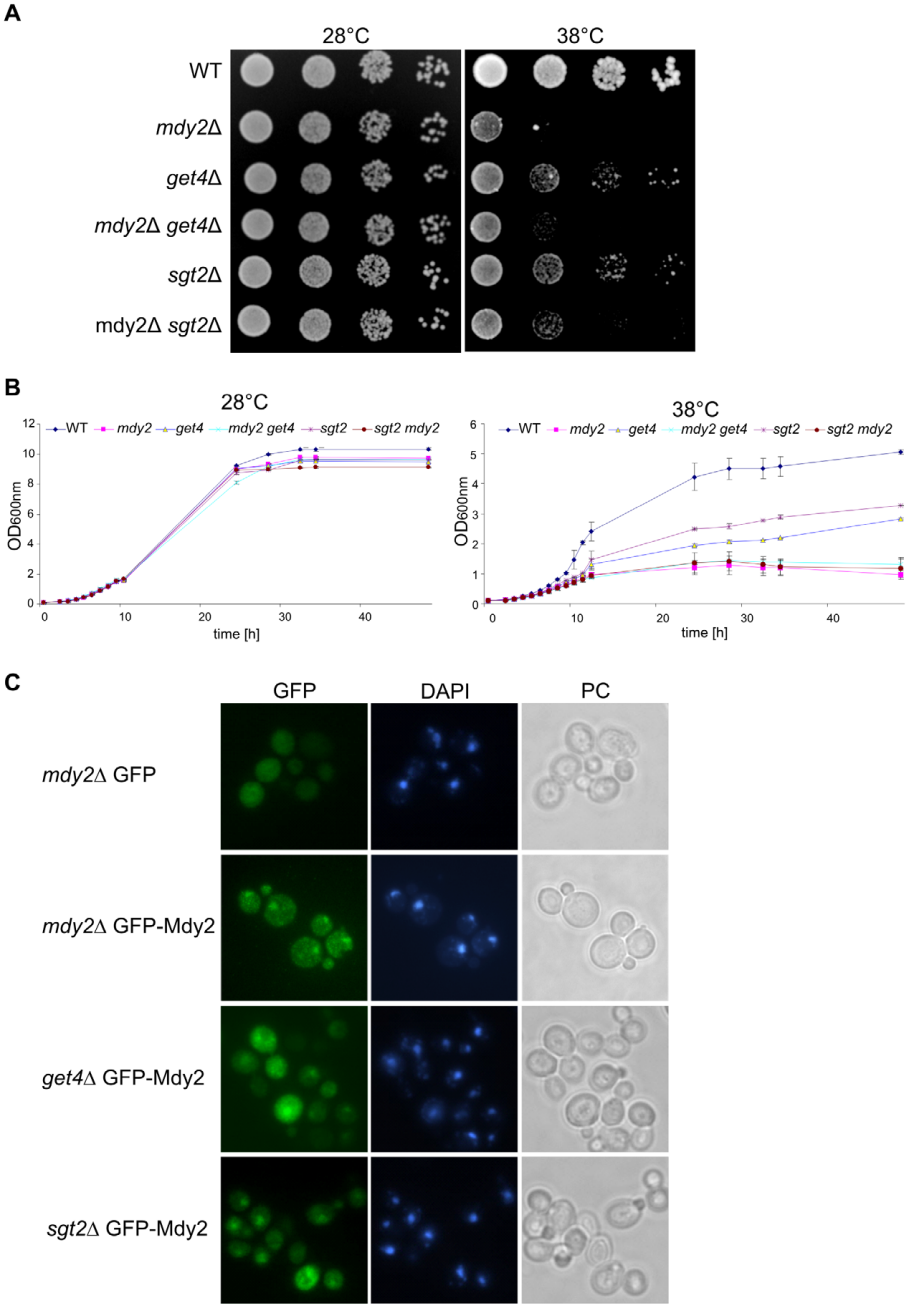
Deletion of *GET4* or *SGT2* does not modify heat sensitivity or nuclear localization of Mdy2. (A) Temperature sensitivity of *mdy2*Δ cells. Equivalent to 0.4 OD_600_ units of exponentially growing yeast cells diluted serially and spotted on YEPD agar plates. The plates were incubated at 28°C and 38°C for 2 days and pictures were taken. A representative experiment is shown. (B) Temperature sensitivity of *mdy2*Δ cells grown in liquid medium. Experiments were repeated three times with a similar outcome. The error bars represent standard error of the mean. (C) Mdy2 localizes mainly to the nucleus. Mdy2 in *mdy2*Δ, *get4*Δ, and *sgt2*Δ cells was localized by direct fluorescence of exponentially growing yeast cells using green fluorescence protein (GFP) (*green channel*). The mutant *mdy2*Δ was transformed with plasmid MDY2p-GFP-MDY2 (pZH152) or control vector. Nuclear DNA was stained by DAPI to indicate positions of nuclei (*blue channel*). Green fluorescence images of GFP-Mdy2 were recorded by a Zeiss Axioscope fluorescence microscope. Cells are shown by phase-contrast (PC) images and nuclear DNA by DAPA staining. Strains: WT (W303-1A), *mdy2*Δ (HZH686), *get4*Δ (HKA200), *sgt2*Δ (HLS2002), *mdy2*Δ *get4*Δ (HKA227), and *mdy2*Δ *sgt2*Δ (HLS2024).

As mentioned above, Mdy2 function during the assembly of TAPs in ER-membrane as part of the Get4/Sgt2 complex has been studied extensively [22]. To analyze the heat-induced stress tolerance we constructed single and double deletions of *GET4* and *SGT2* with *MDY2* and tested them under heat stress. Exponentially growing cells were serially diluted and dropped on selective plates and incubated at 28°C and 38°C. Consistent with previous results *mdy2*Δ strains exhibit reduced stress tolerance at elevated temperature (Figure 1A). In contrast *GET4* or *SGT2* deletion showed only weak heat-sensitivity in comparison to wild type strains. Double deletion strains of *mdy2*Δ*get4*Δ or *mdy2*Δ*sgt2*Δ exhibit no synergistic sensitivity (Figure 1A). We also addressed the ability of *mdy2*Δ, *get4*Δ and *sgt2*Δ strains to grow in liquid medium at 38°C (Fig. 1B). In agreement with the reported phenotype on plate assays, growth in liquid medium at 38°C supports the notion that *MDY2* deletion mutants increase temperature sensitivity of the yeast cells. *GET4* and *SGT2* deletion mutants showed only weak heat-sensitivity in comparison to wild type strains.

Next, we analyzed nuclear localization of Mdy2 in the absence of Get4 or Sgt2. Consistent with previous results, GFP-Mdy2 predominantly accumulates in the nucleus. The same is true for *get4*Δ as well as *sgt2*Δ strains (Figure 1C), suggesting that a functional Mdy2/Get4/Sgt2 complex is not essential for nuclear localization of Mdy2 (Figure 1C). In summary, genetic and cell biological data support the assumption that Mdy2 regulation of heat-induced stress tolerance is independent of Get4 and Sgt2 and thus, most likely, a novel function of the Mdy2.

### The Fungal-specific N-terminus of Mdy2 is Functionally Important during Heat Stress

**Figure 2:**
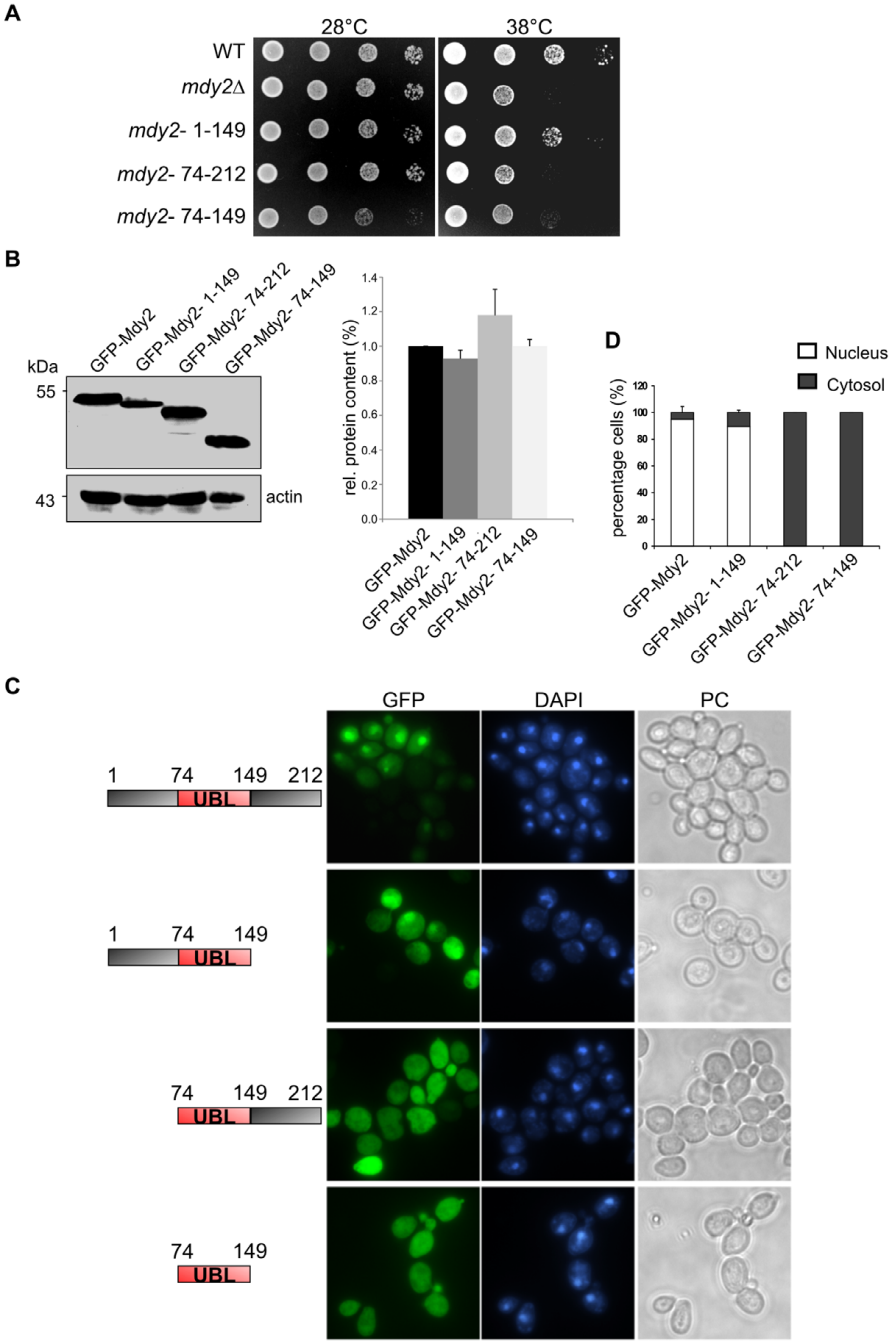
Deletion of the N-terminal region Mdy2 affects GFP-Mdy2 nuclear localization and heat sensitivity. (A) Mdy2 and different C-, N-, and NC-terminal deletion fragments of Mdy2 open reading frame (see schematics) fused to the C-terminus of GFP protein expressed under the control of *GAL1* promoter in *mdy2*Δ (HZH686) cells. Temperature sensitivity recorded as indicated in Figure 1A. Representative experiments are shown. (B) Protein expression level of GFP-Mdy2 variants shows no difference in mutant cells. The left panel shows Western blot of total protein extracts from GFP-Mdy2, GFP-Mdy2- 1–149, GFP-Mdy2- 74–212, and GFP-Mdy2- 74–149 expressing yeast cells. GFP-Mdy2 was detected using anti-GFP antibody. Protein expression of actin as internal standard was performed using anti-actin antibody, clone C4/MAB1501 (left panel). Quantitative densitometry of protein expression showed no changes in the protein levels of GFP-Mdy2 variants. GFP-Mdy2 was set to 1 (right panel). (C) Visualization of exponentially growing indicated yeast cells was performed using fluorescence microscopy as in Figure 1B. (D) Quantitative and statistical analysis of the subcellular localization of GFP-Mdy2 variants. About 100 cells from three independent experiments were counted. The graphs show the percentage of cells demonstrating nuclear or cytosolic GFP-Mdy2 variant protein distribution.

Mdy2 is 212 amino acids long. Structural analyses suggest a tripartite domain structure which is common in all fungi (reviewed in [23]). The N-terminal domain of Mdy2 is 73 amino acids long and shows interaction with Get4 [24], followed by the highly conserved UBL domain from amino acid 74 to 149 and the C-terminal domain from amino acid 150 to 212. The latter has been implicated in Mdy2 homodimerization [24], [25]. Sequence similarity between Mdy2 and the mammalian homolog GdX (Ubl4a, 154 amino acids in length) encompass residues 74–212 of Mdy2 and the N-terminal 123 residues of GdX. To map functional domains of Mdy2, various truncated versions were constructed by PCR and *in vivo* recombination (see Material and Methods). After verification corresponding strains were examined in response to heat stress (as above; Figure 2A). Strains expressing Mdy2 and Mdy2ΔC (residues 1–149) showed similar growth behavior suggesting that the C-terminal region is dispensable for heat-induced stress response. However, strains expressing Mdy2ΔN (residues 74–212) or Mdy2UBL (residues 74–149) revealed similar heat sensitivity and growth defects at elevated temperature as *mdy2*Δ mutants. Western blot analysis of Mdy2 variants revealed no significant differences in protein expression levels compared to the control Mdy2 (Figure 2B).

Analyzing the subcellular localization of the Mdy2 variants revealed that the N-terminal 74 amino acid residues are essential for nuclear localization (Figure 2C and D). Full-length and C-terminal truncated Mdy2 showed a predominantly nuclear staining (Figure 2C, two upper panels and Figure 2D), whereas N-terminal truncated and NC-truncated UBL-domain (residues 74–149) showed a cytoplasmic distribution (Figure 2C, two lower panels and Figure 2D). To summarize, the fungal-specific N-terminal region of Mdy2 is responsible for its nuclear import, and nuclear localization correlates with functional importance during heat stress. The C-terminal end that is involved in Mdy2 homodimerization [24], [25] is, on the other hand, dispensable for nuclear targeting and heat-stress response.

### The N-terminal Region of Mdy2 Contains a Functional NLS

**Figure 3:**
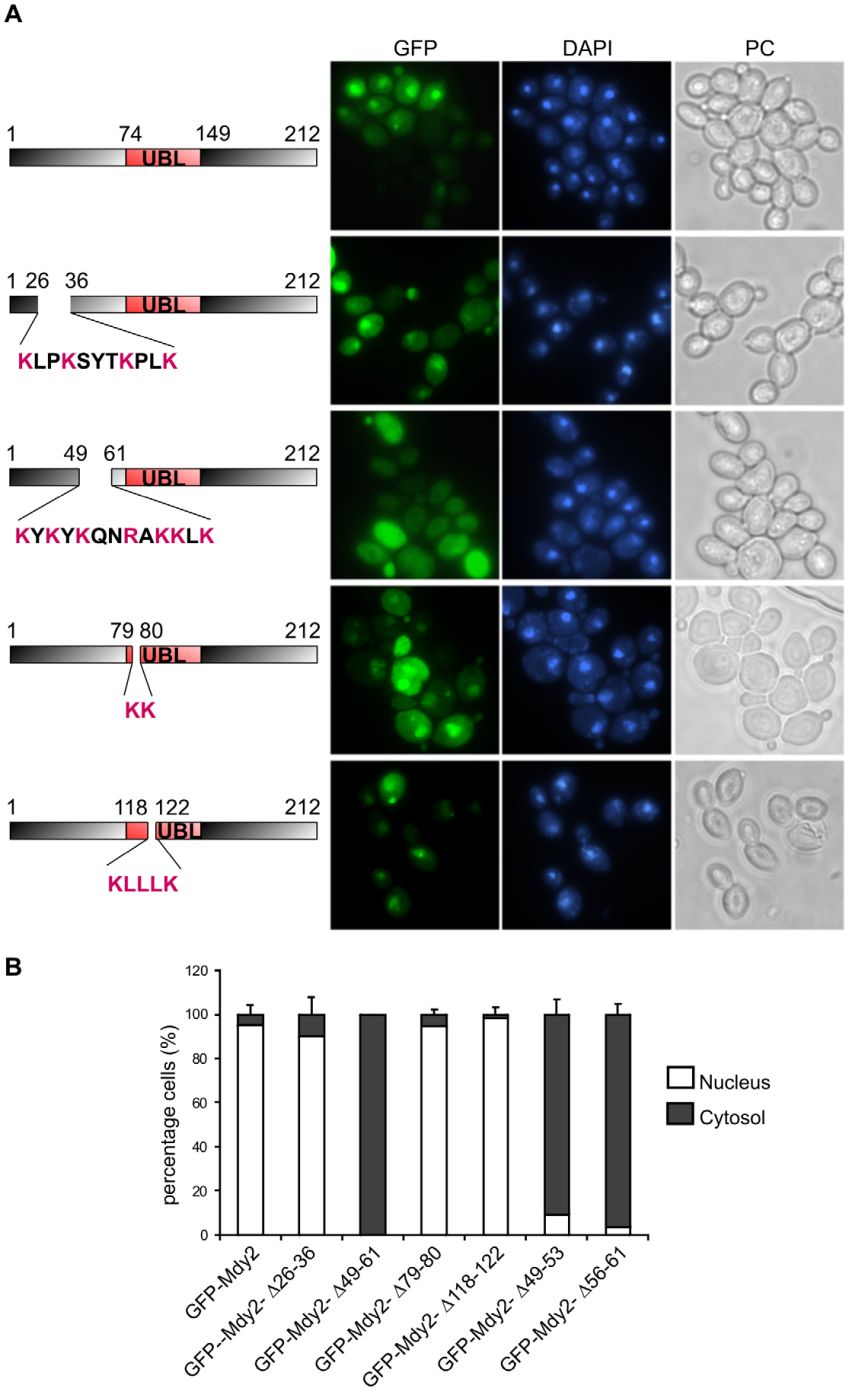
Identification of a nuclear localization signal (NLS) in the N-terminal domain and a nuclear export signal (NES) in the UBL domain of Mdy2. (A) Green fluorescence images and nuclear DNA of exponentially growing *mdy2*Δ cells carrying wild type Mdy2 (*MDY2*) and different putative NLS deletion constructs of Mdy2 (*mdy2*-Δ26–36, *mdy2*-Δ49–61, and *mdy2*-Δ79–80, respectively) were recorded as in Figure 1C. The NLS sequence of Mdy2 is localized between the amino acids 49 and 61 (panel 3). A putative NES deletion construct of Mdy2 (see schematic) was fused to the C-terminus of GFP protein, expressed under the control of *GAL1* promoter in *mdy2*Δ (HZH686) cells, and analyzed as in Figure 1B. The NES sequence of Mdy2 is localized between amino acids 118 and 122 (panel 5). (B) Quantitative and statistical analysis of the subcellular localization of Mdy2 mutants with defective nuclear localization, Mdy2-ΔNLS, and nuclear export, Mdy2-ΔNES as in Figure 2D.

To study nucleocytoplasmic shuttling we searched for a NLS and NES sequences (see below). We identified three potential candidates for classical NLS, which are rich in lysine and arginine (amino acid residues 26–36 KLPKSYTKPLK, 49–61 KYKYKQNRAKKLK and 79–80 KK). Mutant versions of Mdy2 carrying deletions in these regions were generated by assembly PCR and *in vivo* recombination (see Materials and Methods). The correct sequence and expression were verified (Figure S1A and data not shown). We then examined the growth behavior of cells carrying these deletions, Mdy2-Δ26–36, Mdy2-Δ49–61, and Mdy2-Δ79–80, in response to heat stress as described (Figure S1B). Full-length Mdy2 and Mdy2-Δ79–80 showed similar growth behavior at elevated temperature suggesting that the deletion of the two lysines is not functionally important. In contrast, Mdy2-Δ26–36 and Mdy2-Δ49–61 exhibit similar heat sensitivity as *mdy2*Δ mutants suggesting that these regions are essential for heat-induced stress tolerance. Strains harboring GFP-Mdy2-Δ26–36 and GFP-Mdy2-Δ79–80 showed a predominantly nuclear staining (Figure 3A, panels 2 and 4 and 3B). However, strains harboring GFP-Mdy2-Δ49–61 showed only cytoplasmic distribution (Figure 3A, panel 3 and 3B). Thus, a functional NLS of Mdy2 is located between amino acid 49 to 61.

GFP-Mdy2-Δ79–80 showed an enhanced nuclear staining suggesting that these two K residues in the UBL domain are involved in the nuclear export of Mdy2 (see below). In an additional step, the 13 amino acid NLS sequence of the protein (49-KYKYKQNRAKKLK**-**61**)** was subjected to partial deletion to test their influence on localization of the fusion protein. Two additional GFP-Mdy2-constructs with deletions of amino acid residues 49–53 (KYKYK) and 56–61 (RAKKLK) were generated, and strains expressing GFP-Mdy2-Δ49–53 and GFP-Mdy2-Δ56–61 were examined for localization of Mdy2. We observed that both segments are necessary for the NLS activity and nuclear import of Mdy2 (Figure S2). These results indicate two important regions in the N-terminus that are essential for yeast heat-stress induced tolerance. One of them has the characteristic features of a functional NLS.

### Mdy2 Carries a Functional Nuclear Export Signal (NES)

The localization of Mdy2 might be influenced by the presence of a nuclear export signal (NES). We used NetNES 1.1 prediction tool, which predicts leucine-rich nuclear export signals (NES) in eukaryotic proteins [26]. The analysis yielded a single sequence, between amino acids 118–122, which could be considered as a nuclear export sequence (NES). We generated mutant versions of Mdy2 carrying deletions in this region (GFP-MDY2-ΔNES) by assembly PCR and *in vivo* recombination and verified its sequence and expression (Figure S1A and data not shown). GFP-MDY2-Δ118–122 localized predominantly in the nucleus and evidently at nuclear foci (Figure 3A, panel 5, 3B and see below) indicating that the amino acid residues from 118 to 122 are important for nuclear export.

In summary, we have delineated features that are required for nuclear import and export of Mdy2: a classic bipartite nuclear localization signal (cNLS) is present in the N-terminal region of Mdy2 between amino acid residues 49 to 61 and a nuclear export signal (NES) is located proximal to the NLS in the UBL domain of Mdy2 between amino acid residues 118 to 122.

### The NES Motif is Important for Interaction of Mdy2 with Sgt2 but not Get4

**Figure 4:**
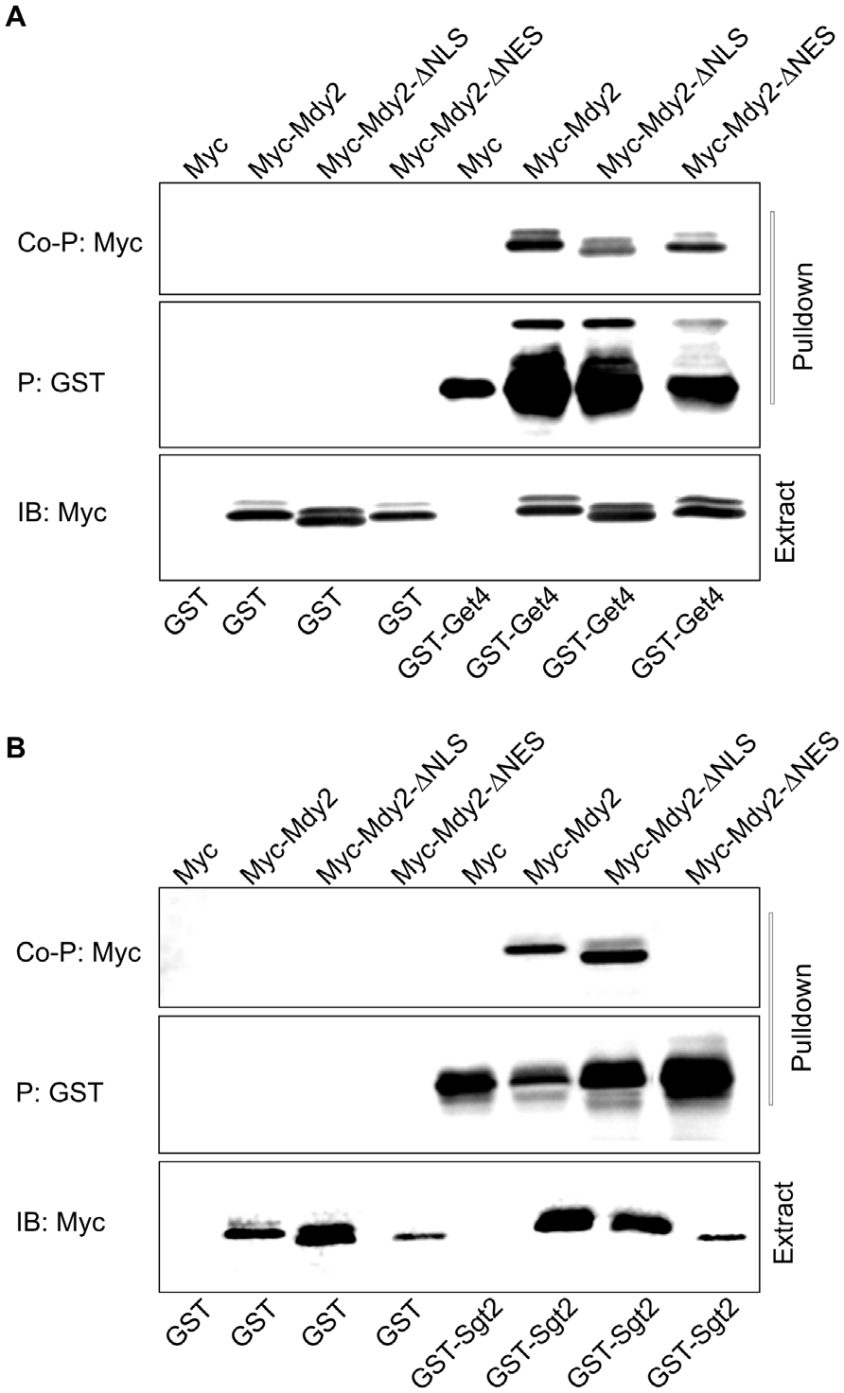
Binding assays of Get4 and Sgt2 with Mdy2-ΔNLS and Mdy2-ΔNES mutant proteins. (A) HZH686 (W303-1A *mdy2*Δ*)* cells were transformed with CEN expression vectors encoding Myc, Myc-tagged Mdy2 (Myc-Mdy2), Myc-Mdy2-NLS, Myc-Mdy2-NES, GST, and GST-tagged Get4 (GST-Get4) under the control of *GAL1* promoter. Cells were grown in SRG to log phase (see Material and Methods), whole cell extracts were prepared and GST-Get4 was precipitated using Glutathione -Sepharose 4B. The pulldown was then tested for the presence of Mdy2 association by probing a Western blot with anti-Myc Ab (top panel). To monitor pulldown recovery, the level of GST-Mdy2 in the binding assay was measured by probing the same membrane with anti-GST antibody (middle panel). Expression levels of Myc-Mdy2 in the whole cell extracts used for binding assay were measured on a Western blot (bottom panel). (B) HZH686 (W303-1A *mdy2*Δ*)* cells were transformed with expression vectors encoding Myc-tagged Mdy2 variants as in (A) and GST-tagged Sgt2 (GST-Sgt2) under the control of *GAL1* promoter. Cells were grown in SRG to log phase, whole cell extracts were prepared, and GST-Sgt2 was precipitated using Glutathione -Sepharose 4B. The presence of Mdy2 in the pulldown was confirmed by probing a Western blot with anti-Myc antibody (top panel). To monitor binding recovery the level of GST-Sgt2 in the pulldown was measured by probing the same membrane with anti-GST Ab (middle panel). Expression levels of Myc-Mdy2 in the whole cell extracts used for pulldown were measured on Western blots (bottom panel).

To test whether deletion of NLS and/or NES affects the association of Mdy2 with the previously identified partner proteins Get4 and Sgt2, co-precipitation experiments were conducted. In accord with previous results [27], [28], GST-tagged Get4 or Sgt2 can bind Mdy2 (Figure 4A and 4B, lane 6). Similarly, GST-tagged Get4 or Sgt2 can interact with Mdy2-ΔNLS (Figure 4A and 4B, lane 7) indicating that these 13 amino acids from the N-terminal region of Mdy2 have no effect on the interaction of Mdy2 with Get4. However, deletion of the 4 amino acid long NES motif within the UBL domain (MDY2-ΔNES) abolishes binding of Mdy2 to Sgt2 (Figure 4B, lane 8). From this assay and in accord with previous results [28], we concluded that the NES in the UBL domain of Mdy2 is the predominant interaction surface of Mdy2 for Sgt2 which might function in masking this export signal.

### Nuclear Import of Mdy2 is Necessary for Heat Stress-induced Response and Recruitment to Cytoplasmic Stress Granules

**Figure 5:**
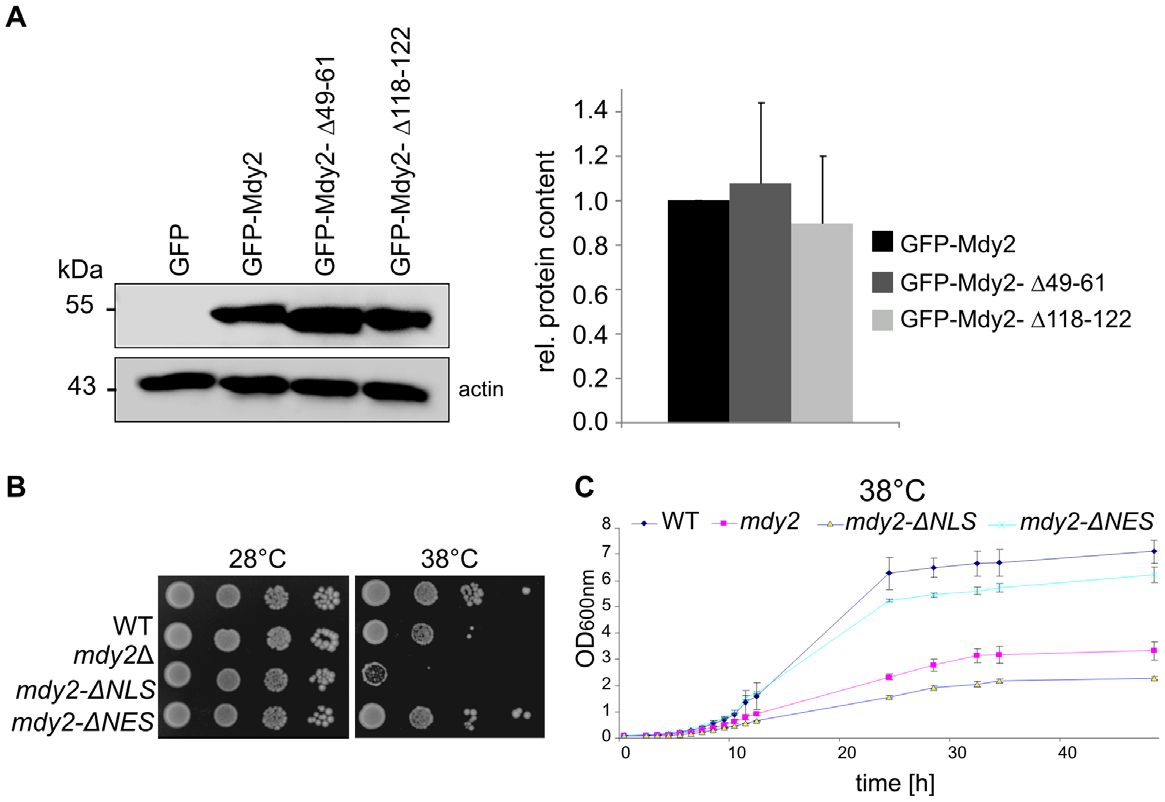
Heat sensitivity of Mdy2 mutants with defective nuclear localization, Mdy2-ΔNLS, and nuclear export, Mdy2-ΔNES. (A) Protein expression level of GFP-Mdy2 and different putative NLS and NES constructs of Mdy2 is equal. Western blotting analysis of NLS and NES deletion constructs (*mdy2-*Δ*NLS* and *mdy2-*Δ*NES*, respectively) in CEN plasmids expressed under the control of *MDY2* promoter in *mdy2*Δ (HZH686) cells (left panel). Quantitative densitometry of protein expression showed no changes in GFP-Mdy2 variants. GFP-Mdy2 was set to 1 (right panel). (B) Temperature sensitivity of *mdy2*Δ cells carrying wild type Mdy2 (*MDY2*), empty vector (*mdy2*Δ), NLS and NES deletion constructs (*mdy2-*Δ*NLS* and *mdy2-*Δ*NES*, respectively) in CEN plasmids expressed under the control of *MDY2* promoter recorded as indicated in Figure 1A. Mdy2 mutants with a defect in nuclear localization *(mdy2-*Δ*NLS)* revealed an enhanced growth defect at elevated temperature (third row). Representative experiments are shown. (C) Temperature sensitivity of *mdy2* mutant cells grown in liquid medium.

To explore the growth of Mdy2-ΔNLS and Mdy2-ΔNES mutants in more detail we studied the growth behavior of cells by expressing *mdy2-*Δ*NLS* (see Figure 3) and *mdy2-*Δ*NES* controlled by the natural *MDY2* promoter sequence (Figure 5). Western blot analysis of Mdy2 variants revealed no significant differences in protein expression levels compared to the control Mdy2 (Figure 5A). *mdy2* mutants with defective nuclear localization (*mdy2-*Δ*NLS*) showed a growth defect at elevated temperatures (Figure 5B, third row). Diversely, *mdy2-*Δ*NES* mutants behaved more or less like wild type (Figure 5B, lower row). We then investigated the ability of *mdy2-*Δ*NLS and mdy2-*Δ*NES* strains to grow in liquid medium at 38°C (Fig. 5C). In agreement with the reported phenotype on plate assays, growth in liquid medium at 38°C supports the notion that *mdy2-*Δ*NLS* showed a growth defect at elevated temperatures. Taken together these results indicate that the NLS but not the NES is essential for the function of Mdy2 in heat-induced stress tolerance.

**Figure 6:**
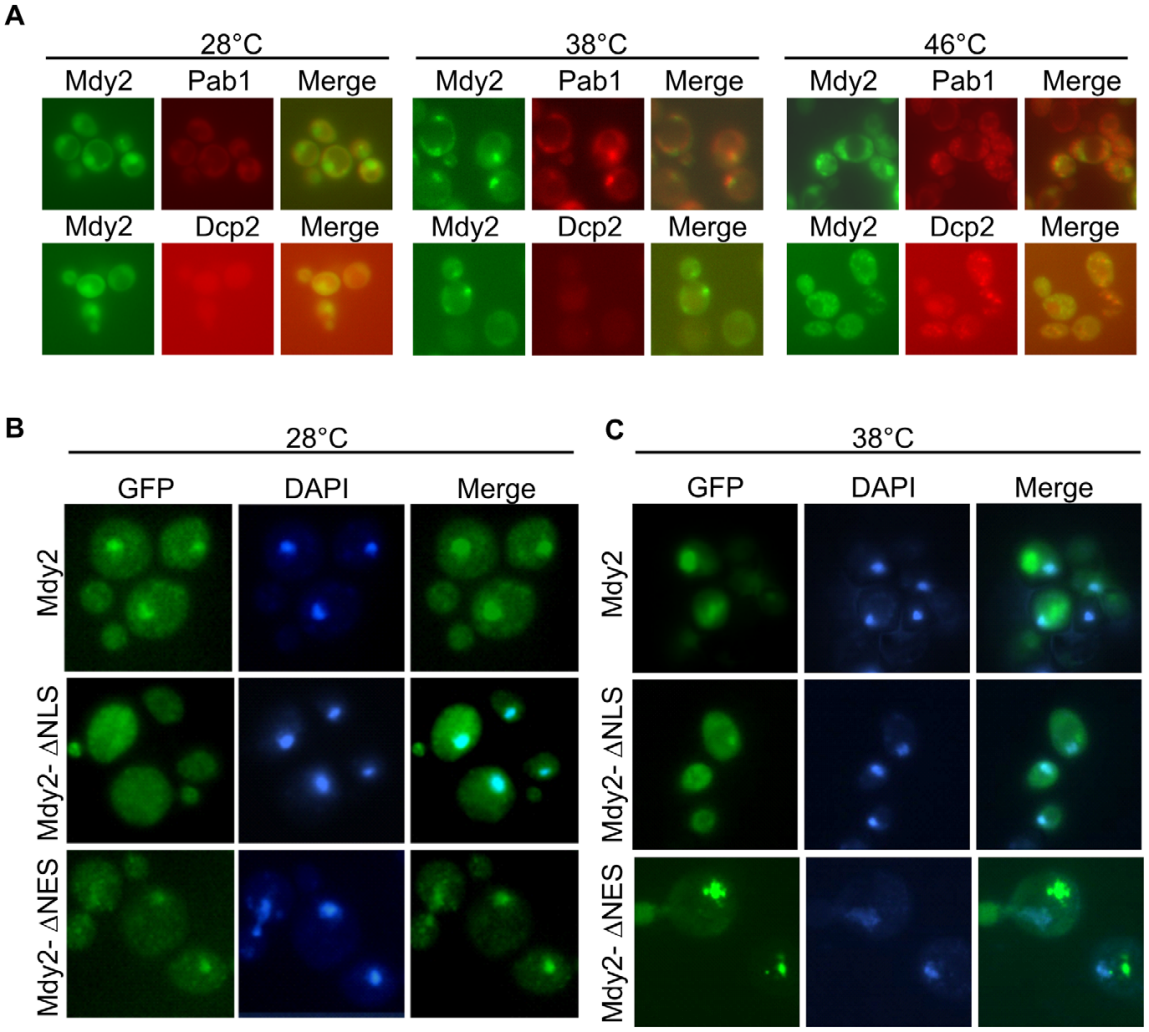
Localization of Mdy2 mutants with defective nuclear localization, Mdy2-ΔNLS, and nuclear export, Mdy2-ΔNES. (**A**) Mdy2 localizes mainly to the nucleus at 28°C. Mdy2 localize to cytoplasmic granules following heat stress. Mdy2 co-localize with Pab1 following mild and robust heat stress. GFP-Mdy2 and Pab1-RFP was visualized by fluorescence microscopy in a *mdy2*Δ strain transformed with plasmids containing GFP-Mdy2 and Pab1-RFP (upper panel) or GFP-Mdy2 and Dcp2-RFP (lower panel) after a temperature shift to 38°C (middle panel) and 46°C (right panel). (B) Localization of Mdy2-ΔNLS and Mdy2-ΔNES mutant proteins at permissive temperature. Exponentially growing *mdy2*Δ cells carrying wild type Mdy2 (*MDY2*), empty vector (*mdy*), NLS and NES deletion constructs (*mdy2-*Δ*NLS* and *mdy2-*Δ*NES*, respectively) fused to the C-terminus of GFP protein in CEN plasmids and expressed under the control of *MDY2*-own promoter. GFP-fusion proteins were localized by direct fluorescence and nuclear DNA was stained by DAPI. (C) Localization of Mdy2-ΔNLS and Mdy2-ΔNES mutant proteins during heat shock-induced stress. Localization pattern for Mdy2-ΔNLS and Mdy2-ΔNES mutant proteins as indicated in (A) during heat shock-induced stress. Localization of GFP-Mdy2 and GFP-Mdy2-NES to the cytoplasmic granules following heat stress is shown in upper and lower panel.

GFP-Mdy2 proteins which are localized predominantly in the nucleus at permissive temperature are localized to cytoplasmic foci during heat shock and glucose deprivation [4]. Co-localization studies revealed that mild heat stress-induced enrichment of Mdy2 in cytoplasmic foci merged mainly with stress granules marker Pab1 ([4] and Figure 6A, middle panel). Accordingly, shifting wild-type cells to either 37°C or 39°C led to a modest and reproducible increase in the number and size of stress granules and P-bodies [29]. During robust heat stress, and in accord with previous studies [30], Mdy2 foci overlapped with P-bodies marker Dcp2 and with SG marker Pab1 (Figure 6A, right panel).

**Figure 7:**
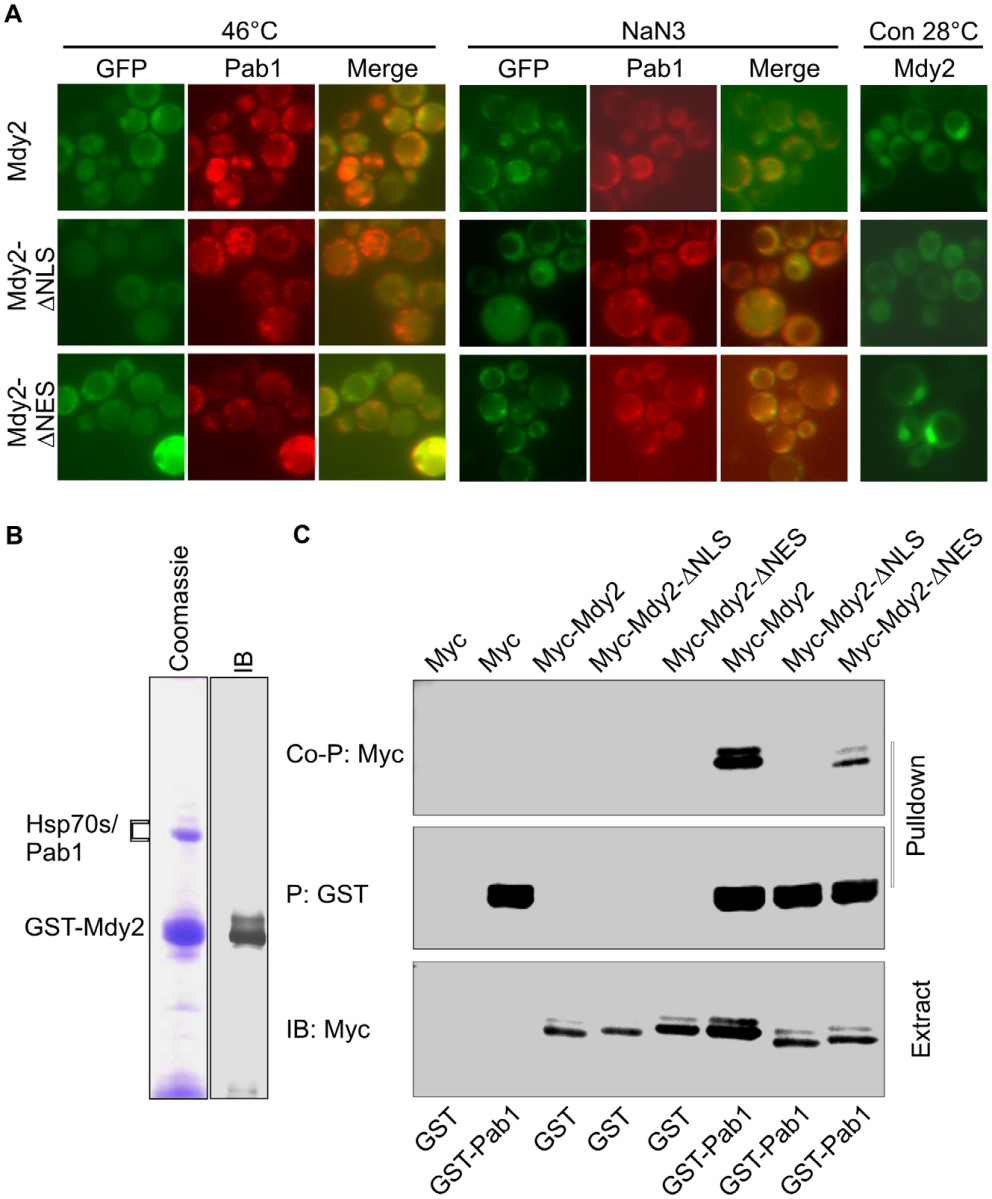
Mdy2 co-localize and interact with Pab1. (A) Mdy2 co-localize with Pab1 following heat stress and treatment with sodium azide. GFP-Mdy2 and Pab1-RFP was visualized by fluorescence microscopy in a *mdy2*Δ strain transformed with plasmids containing GFP-Mdy2 (upper row), GFP-Mdy2-ΔNLS (middle row) or GFP-Mdy2-ΔNES (lower panel), and Pab1-RFP, after a temperature shift to 46°C (left panel) and after treatment with sodium azide (NaN_3_) (right panel). In the overlay pictures (merge), overlap of the colors appears yellow. GFP-Mdy2 and GFP-Mdy2-ΔNES but not GFP-Mdy2-ΔNLS are predominantly nuclear in control (Con) conditions at 28°C (right panel). (B) Mdy2 interacts with Pab1. Cell lysates from the GST-tagged Mdy2 strains were precipitated (P) with Glutathione Sepharose 4B. Following washing, the resin was eluted with glutathione. Eluted proteins were resolved by SDS-PAGE and visualized by immunobloting (control, IB) and Coomassie blue staining (Coomassie). Protein identities were established by mass spectrometry analysis. (C) Extracts from yeast strains HZH686 (W303-1A *mdy2*Δ*)* coexpressing GST alone (GST) or GST-tagged Pab1 (GST-Pab1) with Myc alone (Myc), Myc-tagged Mdy2 (Myc-Mdy2), Myc-tagged Mdy2-ΔNLS (Myc-Mdy2-ΔNLS) or Myc-tagged Mdy2-ΔNES (Myc-Mdy2-ΔNES) were subjected to pulldown using Glutathione Sepharose 4B as in Figure 4. The coprecipitation of indicated Myc-tagged Mdy2 proteins in the pulldown was confirmed by probing a Western blot with anti-Myc Ab (top panel, Co-P: Myc). To monitor pulldown recovery, the level of GST-Pab1 in the pulldown was measured by probing the same membrane with anti-GST Ab (second panel from the top, P: GST). Expression levels of indicated Myc-tagged Mdy2 proteins and GST-Pab1 in whole cell extracts (Extract) used for pulldown were measured on Western blots (third and fourth panels from top, IB:Myc and IB:GST, respectively).

We then addressed the important aspect of subcellular localization of Mdy2 versions under heat stress conditions. As shown before, GFP-Mdy2-ΔNLS protein is distributed in the cytoplasm, whereas the GFP-Mdy2-ΔNES accumulated predominantly in the nucleus at permissive temperatures (Figures 3, 6B and 7A). During mild heat stress GFP-Mdy2 proteins are targeted from a predominantly nuclear position to cytoplasmic foci (Figure 6A, middle panel and Figure 6C, upper panel; [4]). Analyzing Mdy2-ΔNES revealed that this mutant version, like wild-type, also accumulates in cytoplasmic foci and hardly in the nucleus (Figure 6C, lower panel). We then examined subcellular distribution of Mdy2, Mdy2-ΔNLS and Mdy2-ΔNES in stress conditions such as robust heat stress (46°C) [30] and treatment with 0.5% (v/v) sodium azide, which also induces stress granules-like foci [29]. We could verify co-localization of GFP-Mdy2 and GFP-Mdy2-ΔNES with Pab1 under robust heat stress and NaN_3_ treatment conditions (Figure 7A) Thus, apparently during heat stress an NES sequence-independent signal results in nuclear export and cytoplasmic accumulation.

Interestingly, analysis of Mdy2-ΔNLS revealed that this version, although present in the cytoplasm, is unable to accumulate in cytoplasmic foci (Figure 6C, and 7A). This suggests that the nuclear history of Mdy2 is important for function during heat stress and for recruitment to cytoplasmic Pab1-positive stress granules (see below).

### Nuclear Imported Mdy2 Physically Interacts with Pab1

During affinity purification of GST-Mdy2 we found distinct and reproducible protein bands around 70 kD (Figure 7B). Analyses of isolated complex by mass spectrometry resulted in the identification of three Hsp70 proteins, Ssa1, Ssa2, Ssb1, and poly(A)-binding protein Pab1 as the highest scoring proteins. The same analysis allowed the identification of four other proteins but with much lower scores: pre-mRNA polyadenylation factor Fip1, checkpoint serine/threonine-protein kinase Bub1, transketolase Tkl1, and ATP-dependent RNA helicase Dbp3. Previous studies showed that Mdy2 exits in complex with Hsp70s [11]. But the most interesting finding in this experiment is that three of these 8 identified proteins, Pab1, Fip1, and Dbp3 are components of the mRNA processing complex. Of these, Pab1 was the most interesting candidate because it showed a high score, like, Hsp70s in complex with Mdy2 and is known to co-localize with Mdy2 [4]. Here we checked the interaction between Mdy2 and Pab1 in reciprocal co-precipitation experiments and we extended this approach to monitor Pab1 co-precipitation with Mdy2-ΔNLS and Myc-Mdy2-ΔNES (Figure 7C). We confirmed the interaction between Mdy2 and Pab1 as we detected Myc-tagged Mdy2 from yeast cells expressing a GST-Pab1 fusion (Figure 7C, lane 6). We found that Myc-tagged Mdy2 and, to a lower extent, Myc-Mdy2-ΔNES were co-precipitated with GST-Pab1 (Figure 7C, lane 6 and 8). However, Myc-Mdy2-ΔNLS abolishes binding of Mdy2 to Pab1 (Figure 7C, lane 7) indicating that Myc-Mdy2-ΔNLS cannot bind to GST-Pab1. Hence it is most likely that Mdy2 already interacts with Pab1-containing messenger ribonucleoprotein (mRNP) complex in the nucleus. This interaction appears to be important for the recruitment to stress granules and this, again, correlates with the heat stress function of Mdy2.

## Conclusions and Perspectives

Altogether, nucleocytoplasmic shuttling correlates with the heat stress function of Mdy2 and the accumulation of Mdy2 in stress granules. Cytoplasmic stress granules in *S. cerevisiae* contain proteins like Pab1 [31], [32]. Importantly, Mdy2 not only interacts with Pab1 but also co-localizes with Pab1 following heat stress [4]. Pab1 is the major poly(A)-binding protein in yeast. It is a multifunctional protein that mediates many cellular functions associated with the 3′-poly(A)-tail of messenger RNAs. Pab1 shuttles between the nucleus and the cytoplasm and functions in mRNA export [33]. Pab1 therefore appears to actively enter the nucleus and exit via two transports pathways: one pathway is dependent on XPO1/CRM1 through an NES located at the amino terminus of Pab1, whereas the second pathway requires MEX67 and/or ongoing mRNA export [33]. In analogy with Pab1 nuclear export Mdy2 carries a functional NES, but during heat stress an NES sequence-independent signal results in nuclear export and accumulation in cytoplasmic foci. Taking into account our results and a list of Mdy2 interactors like heat shock proteins and Pab1 (this paper and [4], [11], [34], [35]) we come to the conclusion that Mdy2 might play a role in mRNA metabolism. Interestingly, also the human ubiquitin-like protein GdX (Ubl4a) interacts with similar proteins [36], [37] suggesting an evolutionarily conserved process.

In response to stress, eukaryotic cells reprogram the mRNA metabolism to repair stress-induced damage and adapt to changed conditions. During this process the translation of mRNAs encoding “housekeeping” proteins is aborted, whereas the translation of mRNAs encoding molecular chaperones and enzymes involved in damage repair is enhanced. Selective recruitment of specific mRNA transcripts into stress granules is thought to regulate their stability and translation [38]. Heat stress granules selectively exclude mRNAs encoding stress-induced heat shock proteins [39]. In *S. cerevisiae* heat shock induces the formation of stress granules that contain translation initiation factors and non-heat shock mRNAs capable of redistributing into the cytoplasm, and presumably reengaging in translation, upon recovery [30], [40]. The robust heat shock-induced stress granules also contain a subset of processing body components involved in RNA degradation including Dcp2 and Dhh1, yet are spatially distinct from other processing body markers [30]. The fact that heat stress triggers stress granule assembly but not processing body formation indicates that these processes are regulated by distinct signaling pathways [29], [30], [41]. It has been proposed that stress granules are sites where the increased local concentration of proteins and mRNA allows for remodeling and redistribution of mRNPs [40]. Alternatively, specific proteins might be selectively sequestered into or away from stress granules, thus affecting biochemical processes in the cell. For example, recruitment of specific proteins such as RACK1, which is required to activate the apoptosis-inducing MTK1 kinase during stress, to stress granules can inhibit apoptosis [42].

Our results are consistent with the hypothesis that stress granule formation is one of the immediate protective mechanisms against heat stress. We speculate that Mdy2-containing stress granules fulfill a protective role for cell survival upon stress. Under these conditions, Mdy2 might accompany the Pab1-containing mRNP complex to stress granules, and this appears to be functionally important for stress protection. The mechanism and function of Mdy2 in the process remains unclear, but it appears to be essential for a rapid cellular response to stress in processes like stress granules dynamics in the control of non-translating mRNPs. This constitutes a novel link between UBL proteins and posttranscriptional processes.

## Materials and Methods

### Yeast Strains, Media, and Standard Methods

Recombinant DNA techniques were applied according to standard protocols. Depending on the experimental context, yeast cells were grown either in YEPD medium (2% glucose, 2% peptone, 1% yeast extract), synthetic complete medium (0.67% yeast nitrogen base without amino acids) with 2% glucose (SD) containing the required nutrient supplements, or in synthetic raffinose galactose (SRG) medium (0.67% yeast nitrogen base without amino acids, 3% raffinose, 1% galactose) containing the appropriate nutrient supplements. All strains used in this study are in W303-1A (*MAT* ***a*** *leu2-3,112 ura3-1 trp1 his3-11 ade2 can1-100*) background (source: R. Rothstein, Columbia University, New York). HZH686 (W303-1A *MAT* ***a*** *mdy2*Δ*::KanMX4*) was previously described [2]; HKA200 (W303-1A*MAT* ***a*** *get4*Δ*::HIS3),* HKA227 *(*W303-1A *MAT* ***a*** *mdy2*Δ*::KanMX4 get4*Δ*::HIS3*), HLS2002 (W303-1A *MAT* ***a*** *sgt2*Δ*::HIS3*), and HLS2024 *(*W303-1A *MAT* ***a*** *mdy2*Δ*::KanMX4 sgt2*Δ*::HIS3*) are constructed by homolog recombination. Expression vectors encoding full-length truncated or mutated Mdy2, as well as Get4 and Sgt2 were produced by PCR or assembly PCR, accordingly, and cloned by *in vivo* recombination into the galactose-inducible pGREGs expression vectors [43] or in vectors with the appropriate endogenous promoter and checked by restriction digestion, PCR, sequencing, and Western blot analysis. Plasmid MDY2p-GFP-MDY2 (pZH152) was previously described [2]. Plasmid pPAB1-RFP (PAB1-RFP LEU2, CEN) is a kind gift of Charles N. Cole (Dartmouth Medical School). Plasmids pRP1155 (pDCP2-RFP LEU2, CEN) is a kind gift of Roy Parker (University of Arizona).

### Gene Deletion and PCR-mediated Gene Tagging

Deletion strains were constructed by homologous recombination with appropriate PCR-derived disruption cassettes. Each of these consisting of a central marker gene (*KanMX6* or *HIS3MX6*) flanked by sequences homologous to regions adjacent to the gene to be disrupted. The disruption cassettes were introduced into cells by the high-efficiency LiAc transformation method. Correct disruption was verified by analytical PCR.

PCR-mediated gene tagging was performed using a similar method. The integration cassettes used consisted of a central tag (GST, Myc or *GFP*) with a selection marker (*HIS3*, *TRP1* or *KanMX6*) and flanking sequences homologous to 3′-terminal sequences of the gene to be tagged. Correct tagging was verified by analytical PCR and sequence and expression analysis.

### Immunoblot Analysis

For expression controls, log-phase yeast cells (equivalent to 4 OD_600_ units) were harvested by centrifugation (5 min, 3000 rpm), washed once in E-buffer (20 mM HEPES pH 7.5, 150 mM NaCl, 5 mM EDTA pH 8, 0.1% Triton-X-100, 10% glycerol), then resuspended in lysis buffer (E-buffer +1 mM DTT, 0.1 mM Pi-Mix [pepstatin and aprotinin (at 1 mg/ml each), leupeptin and antipain (at 5 mg/ml each), 0.1 mg/ml trypsin inhibitor, and 1 mM PMSF]. Cells were broken down with (2/3 vol) glass beads and collected by centrifugation (2 min, 3000 rpm). The samples were denatured in SDS-PAGE sample buffer by boiling (10 min at 95°C), centrifuged briefly, applied to a 7.5% or 10% SDS-polyacrylamide gel and fractionated by electrophoresis. The proteins were then blotted onto a nitrocellulose membrane according to standard protocol, and probed with polyclonal rabbit anti-GST (1∶1000) and/or monoclonal mouse anti-Myc (1∶1000). Bound primary antibodies were detected enzymatically with goat anti-rabbit and goat anti-mouse antibodies coupled to alkaline phosphatase or horseradish peroxidase (1∶5000 and 1∶50.000, respectively).

### Binding Assay

Cells to be used for binding assays were induced for 16 h in SRG medium, and extracts (equivalent to approximately 12 OD_600_ units) were prepared as described above. 20 µl Glutathione Sepharose 4B (GE Healthcare, Germany) were added to 1 ml of extract and the samples were incubated for 2 h at 4°C. Then the samples were centrifuged (3 min, 13,000 rpm) and the pelleted complexes were washed four times in E-buffer+(see above). The samples were then boiled, fractionated on a 7.5% SDS-polyacrylamide gel and subjected to immunoblotting analysis as described above.

### Microscopy

Cells were grown to log phase in YPD or in appropriate selection medium. Samples (equivalent to 1.5 ml of cells) were taken and washed twice with 500 µl of PBS (pH 7.0) in 1.5 ml reaction tubes. The cells were fixed for 5 min in 70% ethanol and again washed twice in PBS (pH 7.0). To stain DNA, cells were incubated for 10 min in 20 µl of DAPI (1 µg/ml) and washed twice in PBS (pH 7.0). Cells containing GFP-labeled Mdy2 or Mdy2 mutants were used for localization of fusion proteins. Images were acquired by fluorescence microscopy using a Zeiss Axioscope and Zeiss filter systems.

## Supporting Information

Figure S1 **Analysis of a nuclear localization signal (NLS) in the N-terminal domain and a nuclear export signal (NES) in the UBL domain of Mdy2.** (A) Protein expression levels of GFP-Mdy2 and different putative NLS and NES constructs of Mdy2 show no difference in mutant cells. Putative NLS deletion constructs of Mdy2 and a putative NLS deletion construct of Mdy2 (see schematics in Figure 6) were fused to the C-terminus of GFP protein and expressed under the control of the *GAL1* promoter in *mdy2*Δ (HZH686) cells. Western blot analysis (left panel) and quantitative densitometry of protein expression (right panel) showed no changes in protein level of GFP-Mdy2 variants. GFP-Mdy2 was set to 1. (B) Temperature sensitivity of *mdy2*Δ cells carrying wild type Mdy2 (*MDY2*), empty vector (*mdy2*Δ), and different putative NLS deletion constructs of Mdy2 (*mdy2*-Δ26–36, *mdy2*-Δ49–61, and *mdy2*-Δ79–81, respectively) was analyzed as in Figure 1A. Representative experiments are shown.
(TIF)

Figure S2 **Effect of the mutations on the nucleocytoplasmic distribution of Mdy2.** *mdy2*-ΔNLS mutation constructs of *mdy2*-Δ49–53 and Δ56–61 show cytoplasmic distributions. *mdy2*-Δ49–53 and Δ56–61 deletion construct of Mdy2 was fused to the C-terminus of GFP protein, expressed under the control of the *GAL1* promoter in *mdy2*Δ (HZH686) cells and was analyzed as in Figure 1B. Both Mdy2-ΔNLS mutant proteins show the cytolasmic distributions.
(TIF)
