# Supplementary figures and images for: Nuclear Import of UBL-Domain Protein Mdy2 Is Required for Heat-Induced Stress Response in Saccharomyces cerevisiae
Source: PLoS One. 2012 Dec 28;7(12):e52956. doi: 10.1371/journal.pone.0052956 (PMC3532209; doi:10.1371/journal.pone.0052956)

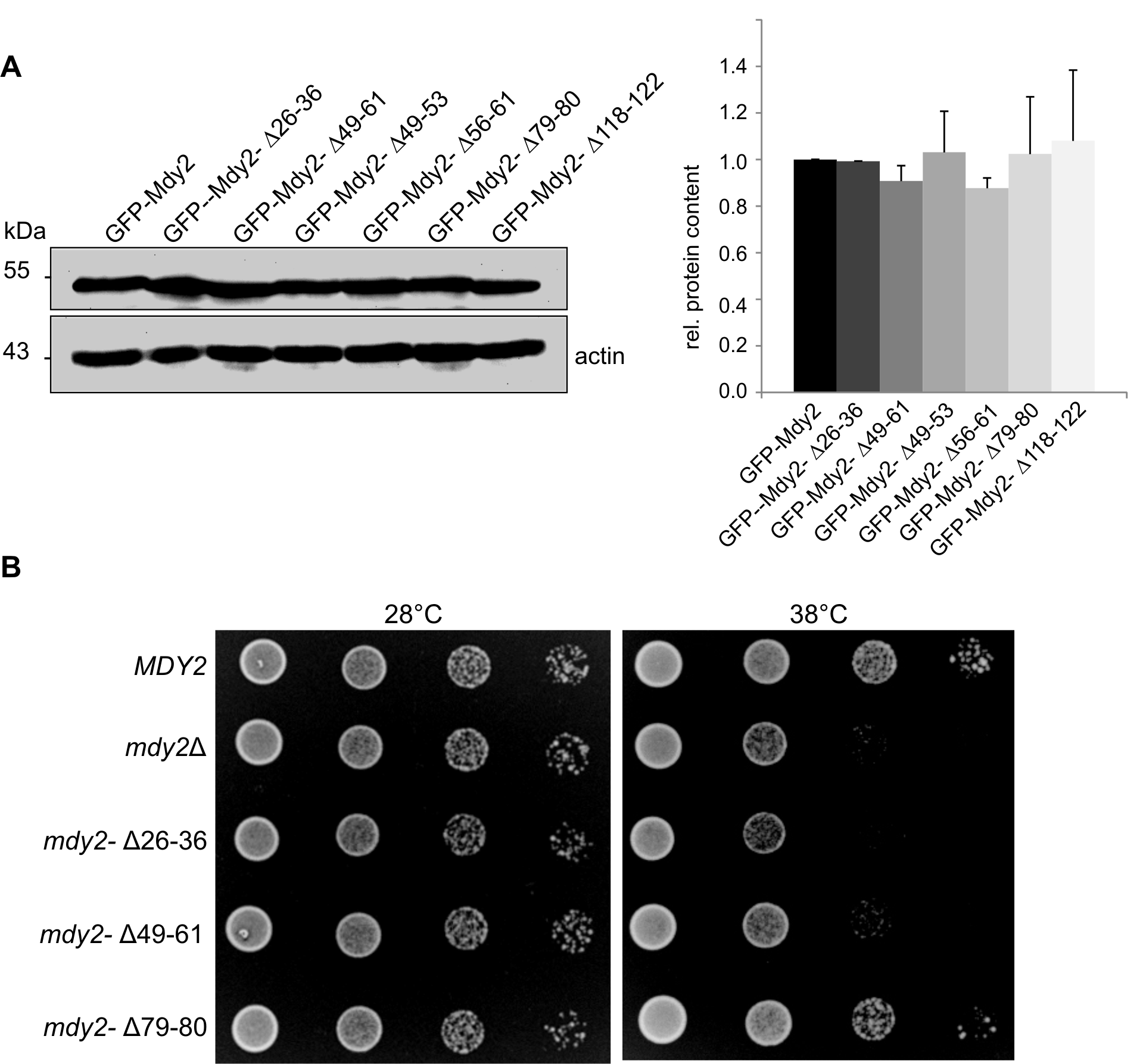

Supplement: Figure S1 — Analysis of a nuclear localization signal (NLS) in the N-terminal domain and a nuclear export signal (NES) in the UBL domain of Mdy2. (A) Protein expression levels of GFP-Mdy2 and different putative NLS and NES constructs of Mdy2 show no difference in mutant cells. Putative NLS deletion constructs of Mdy2 and a putative NLS deletion construct of Mdy2 (see schematics in Figure 6) were fused to the C-terminus of GFP protein and expressed under the control of the GAL1 promoter in mdy2Δ (HZH686) cells. Western blot analysis (left panel) and quantitative densitometry of protein expression (right panel) showed no changes in protein level of GFP-Mdy2 variants. GFP-Mdy2 was set to 1. (B) Temperature sensitivity of mdy2Δ cells carrying wild type Mdy2 (MDY2), empty vector (mdy2Δ), and different putative NLS deletion constructs of Mdy2 (mdy2-Δ26–36, mdy2-Δ49–61, and mdy2-Δ79–81, respectively) was analyzed as in Figure 1A. Representative experiments are shown. (TIF) [file pone.0052956.s001.tif]

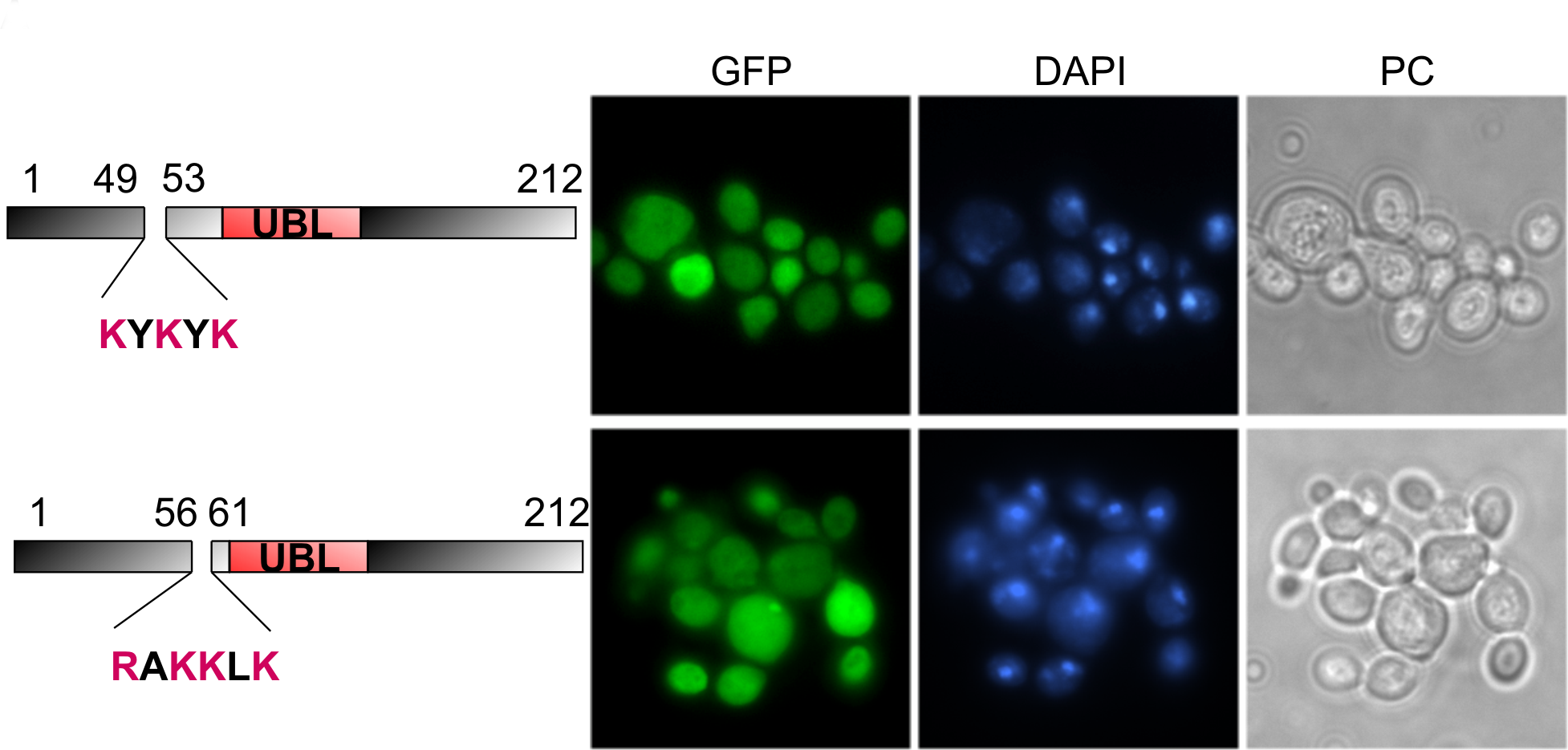

Supplement: Figure S2 — Effect of the mutations on the nucleocytoplasmic distribution of Mdy2. mdy2-ΔNLS mutation constructs of mdy2-Δ49–53 and Δ56–61 show cytoplasmic distributions. mdy2-Δ49–53 and Δ56–61 deletion construct of Mdy2 was fused to the C-terminus of GFP protein, expressed under the control of the GAL1 promoter in mdy2Δ (HZH686) cells and was analyzed as in Figure 1B. Both Mdy2-ΔNLS mutant proteins show the cytolasmic distributions. (TIF) [file pone.0052956.s002.tif]
